# Methylene Blue Solid Alginate Gels for Photodynamic Therapy: The Peculiarities of Production and Controlled Release of the Dye

**DOI:** 10.3390/polym16192819

**Published:** 2024-10-05

**Authors:** Anna Solovieva, Alexander Kopylov, Anastasiya Cherkasova, Ilya Shershnev, Vladislav Kaplin, Victoriya Timofeeva, Anastasiya Akovantseva, Marina Savko, Alexander Gulin, Tatyana Zarkhina, Nadezhda Aksenova, Peter Timashev

**Affiliations:** N.N. Semenov Federal Research Center for Chemical Physics, Russian Academy of Sciences, 4 Kosygina Street, 119991 Moscow, Russia; via_cetra@mail.ru (A.K.); anastasiya-cherk@mail.ru (A.C.); piroklas@gmail.com (V.K.); vik.timofeeva@gmail.com (V.T.); akovantseva-a@yandex.ru (A.A.); msavko@mail.ru (M.S.); aleksandr.gulin@phystech.edu (A.G.); zarkhina@mail.ru (T.Z.); naksenova@mail.ru (N.A.); timashev.peter@gmail.com (P.T.)

**Keywords:** calcium alginate, alginic acid, methylene blue, controlled release

## Abstract

The purpose of this work is to establish the influence of the nature of solid alginate gels (alginic acid, AAG; calcium alginate, CAG) and the conditions of methylene blue (MB) introduction to alginate matrices upon its release into aqueous media. MB is an active photosensitizer, which is used in the photodynamic therapy of tumors and purulent wounds. Solid alginate gels based on AAG and CAG were obtained by adding hydrochloric acid and calcium chloride to sodium alginate. The dye was introduced into the matrix either at the stage of gelation or by immersing the gel in an aqueous solution of the dye. It has been shown that the strength of the dye’s attachment to AAG is higher than that of CAG, which leads to a higher rate of MB release from CAG into aqueous media. It has also been shown that, when introduced at the stage of gel formation, MB is released into both the water and buffer solutions. When MB is introduced by gel immersion into an MB solution, the dye may be released only into salt solutions. An alginate gel with immobilized MB can be used as a solid photosensitizing system with the controlled release of the photoactive agent into the wound cavity for photodynamic treatment.

## 1. Introduction

The controlled release of drugs using long-acting medications is commonly utilized in the treatment of chronic diseases, such as cardiovascular, neurological, and oncological conditions, where it is important to maintain a constant concentration of the drug taken over a long period of time [1,2,3]. However, there are particular situations of difficult-to-heal purulent wounds, trophic ulcers, diabetic feet, and complicated burns, where it is necessary to carry out long-term local antibacterial treatment. This occurs, for example, in the treatment of difficult-to-heal purulent wounds of various etiology by the method of photodynamic therapy (PDT) [4,5]. PDT is based on the ability of a number of photosensitizers (PSs) to selectively accumulate in malignant tumors and, as was shown later, in microorganisms (viruses, bacteria, and fungi) [6], which was discovered in the middle of the last century. Under laser radiation energy, photochemical reactions in PS-treated cells and tissues are initiated, releasing reactive oxygen species, primarily, singlet oxygen ^1^O_2_, which destroys pathological cells and tissues. Healing after PDT sessions occurs similar to the natural reparative processes, which allows, if necessary, repeating the treatment procedures multiple times. It is important that PDT is effective in the treatment of local infections caused, among other things, by antibiotic-resistant microorganisms [7], which expands the area of the method’s applicability. Currently, the latter PDT property is especially valuable since the mortality rate from antibiotic-resistant infections is already approaching the level of that during the pre-antibiotic era [8].

New possibilities of PDT, primarily, the progress in the method’s efficiency, the selectivity of PS accumulation by pathogens causing purulent infection, and the exclusion of a secondary infection of the wound cavity [9], are associated with the development of photosensitizing systems (PSSs), in particular, the inclusion of additional biologically active components (growth factors, enzymes) in the system.

Previously, we have shown that the efficiency of early stage wound healing in laboratory animals after PDT procedures with porphyrin photosensitizers (PPSs) increases when using amphiphilic polymers (Pluronics, polyvinylpyrrolidone, PVP) and polysaccharides (chitosan, CHT; sodium alginate, SA) together with PPSs [10,11]. It should be noted that sodium alginate (SA), a sodium salt of alginic acid produced by an alkaline extraction from brown algae, and CHT have their own bactericidal activity [12]. They are sometimes used as wound-healing agents (in wound dressings) or individual polymers in the treatment of small wounds. These polysaccharides are effective carriers for proteolytic enzymes (trypsin) and powder sorbents with antiseptics (chlorhexidine, furagin), accelerating the healing of wounds and burns [13,14,15]. SA is able to stop local bleeding and stimulate the healing of ulcerative lesions of the gastric and intestinal mucosa [16]. Due to the fact that SA does not have antigenicity or allergenicity, it is completely absorbed in the body, stimulates wound healing processes, and is easily combined with medicinal and other physiologically active substances; it is used in the creation of therapeutic dressings or coatings for the treatment of burns, wounds of various origins, trophic ulcers, radiation skin lesions, and bedsores [16,17,18,19]. On the basis of SA, it is possible to obtain hydrogels by different methods, possessing not only biocompatibility and biodegradability, but also a certain mechanical strength, capable of absorbing water-soluble drugs [20,21]. Due to the above properties, SA gels can be used as matrix carriers in systems for the controlled release of PS molecules into damaged tissues during the long-term treatment of difficult-to-heal wounds using the PDT method. The rate of PS release is determined by the supramolecular structure of the carrier and the nature of the PS–matrix bond. Bactericidal alginate coatings should be able to maintain the sterile state of the affected tissue areas between PDT sessions.

The purpose of this work is to create photosensitizing systems for the PDT of difficult-to-heal wounds, trophic ulcers, and bedsores using methylene blue (MB), an ionogenic dye immobilized on sodium alginate hydrogels (AHs). Calcium chloride and hydrochloric acid were used as gelation agents. The main objective was to study the effect of the supramolecular structure of alginate gels and the mechanism of MB binding to the carrier polymer on the kinetics of dye release. Using different options for producing a gel that determines the supramolecular structure of the carrier and varying the method of MB administration are the ways to specifically regulate the rate and amount of dye released into the affected tissues, which allows for controlled PDT therapy sessions.

Moreover, MB can be considered as a model object for studying the release of aromatic water-soluble drugs from the carrier. In this regard, the data on the influence of the carrier structure on the kinetics of MB release into solutions simulating the different media of the gastrointestinal tract will allow for choosing the optimal carrier for water-soluble drugs with an aromatic structure when creating prolonged release drugs for oral administration.

## 2. Materials and Methods

### 2.1. Materials

**Figure 1:**
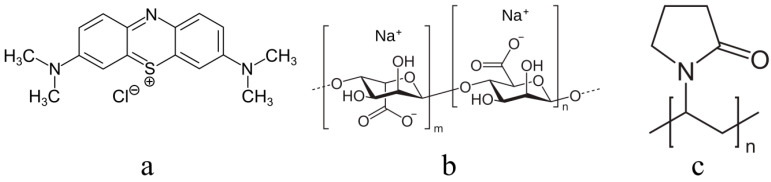
The structural formulas of the reagents: methylene blue (**a**), sodium alginate (**b**), and polyvinylpyrrolidone (**c**).

The structural formula of methylene blue (3,7-bisdimethylaminophenothiazine chloride, Khimmed, Moscow, Russia), a cationic dye used as a photosensitizer in the creation of PSSs for antimicrobial PDT, is shown in Figure 1. MB was introduced into matrices, i.e., gels based on sodium alginate (Ruschem, 150–300 kDa). Calcium chloride (granulated, Ruschem, Moscow, Russia) or hydrochloric acid (Ruschem) were used as gelation agents [22]. The resulting solid polymer gels were used in the form of films (100–300 μm thick). Since it was previously shown that amphiphilic polymers (APs) increased MB activity in the photogeneration of singlet oxygen ^1^O_2_ [23], MB was impregnated into the specified matrices in a solution of polyvinylpyrrolidone (PVP, Aldrich, St. Louis, MO, USA, 40 kDa).

The introduction of methylene blue into alginate gels was carried out either simultaneously with the gelation of the alginate matrix (“mixing” method) or by immersing the alginate hydrogel in a dye solution (“immersion” method).

### 2.2. Mixing Method

Aqueous solutions of MB (C_MB_ = 2 × 10^−5^ M) and MB/PVP (C_MB_ = 2 × 10^−5^ M, C_PVP_ = 5 × 10^−4^ M), were prepared. Solid sodium alginate was dissolved in each solution (C_SA_ = 2 wt.%). The solutions of SA/MB and SA/PVP/MB were divided into two parts: hydrochloric acid (0.1 M) was added to one part of the solution, 5 wt.% aqueous solution of calcium chloride was added to the other one. A hydrogel was formed in the resulting solutions over the course of 24 h, which was then washed with water and dried in air at room temperature until the change in the mass of the samples stopped. The following xerogel films (100–300 μm thick) containing methylene blue were produced: a film of sodium alginate treated with HCl (alginic acid gel, AAG/MB); a film of sodium alginate treated with calcium chloride (calcium alginate gel, CAG/MB); films of gels containing PVP, including AAG/PVP/MB and CAG/PVP/MB.

### 2.3. Immersion Method

A 2 wt.% aqueous solution of sodium alginate was prepared, which was then divided into two parts. One part was treated with hydrochloric acid (0.1 M), the other one was treated with a 5 wt.% aqueous solution of calcium chloride. Hydrogels (in the form of ~200–500 µm thick films) were formed in the treated solutions over the course of 24 h, which were then washed with water. The AAG and CAG hydrogels were immersed in MB and MB/PVP solutions (the concentration of MB and PVP was the same as when introducing the dye into the matrix using the “mixing” method), kept there for 24 h, then removed and dried in air at room temperature until the change in the mass of the samples ceased, forming the corresponding xerogel films (100–300 μm thick). Samples with 0.2–0.9% MB content were used for spectral control of the process kinetics. In the same way, up to 10 wt.% of MB can be introduced to the gels.

### 2.4. Study of the Kinetics of MB Release into the External Medium

The study of the diffusion process of MB from impregnated matrices based on AAG and CAG was carried out as follows: (1) in a hydrochloric acid buffer solution KCl-HCl (pH = 1.6), which was close in acidity to gastric juice, (2) in a phosphate–saline buffer PBS from Eco-Service, Russia (pH = 7.2), simulating the intestinal medium, and (3) in a neutral medium of distilled water (pH = 5.5).

To study the kinetics of MB release into the external medium, a polymer film (~0.2 g) containing MB introduced at the “mixing” or “immersion” stage was placed in a thermostatically controlled cell heated to 37 °C; then, 20 mL of a buffer solution or distilled water was added and stirred with a magnetic stirrer at a speed of 200 rpm. The results of the measurements of the solution’s optical density D (at λ_MB_ = 665 nm) and from characterizing the content of methylene blue released into the buffer solution (KCl-HCl, pH = 1.6; PBS, pH = 7.2) or distilled water during the diffusion from the polymer matrix, were presented as a dependence of the concentrations of methylene blue released from the polymer system at time *t*. The absorption spectra were recorded using a Cary 50 spectrophotometer from Varian (Walnut Creek, CA 94598, USA). The measurement error did not exceed 5%. The amount of the impregnated dye was determined spectrophotometrically by measuring its concentration after the dye was completely released into the solution (from 20 min to 170 h, depending on the method of forming the initial sample).

All dependences demonstrated the first order of the kinetics at the initial stages of the process at time intervals corresponding to the release into solution of 30–50% of the MB amount initially contained in the sample:

C(t) = C_tot_ [1 − exp(−*k*t)], (1)

so that when C(t) << C_tot_.

C(t)/C_tot_~*k*t (2)

The recorded deviations from the first-order kinetics in all cases considered in the study led to errors in the calculated values of the rate constants *k* that did not exceed 5%.

### 2.5. Study of the Structural and Physicochemical Features of the Formed Films

The study of the surface structure and measurement of the local Young’s modulus of alginate gels was carried out using the AFM method. When studying the morphology of the surface areas of hydrogel films, the atomic force microscope (Moscow, Russia, NT-MDT) was used. For comparison, films of the original sodium alginate were used; these films were obtained by the evaporation of a 2% aqueous solution of SA on glass. Etalon probes (Moscow, Russia, NT-MDT) with a nominal stiffness constant of 12 N/m, a nominal resonant frequency of 235 kHz, and a nominal radius of curvature of 10 nm were used. The scans were obtained in the semi-contact mode and 5 to 7 images of 10 × 10 µm were analyzed for each sample. The images were analyzed using Nova software (version 1.0.26.1443) from NT-MDT. The changes in the Young’s modulus values, as a result of treatment with CaCl_2_ or HCl, as well as the effect of MB and PVP on the local stiffness of hydrogels in the liquid state, were studied using a BioScope Resolve atomic force microscope (Bruker, Billerica, MA, USA) in the Force Volume mode. Before the study, the hydrogels were pre-fixed in Petri dishes using cover glasses and filled with distillate. For scanning, a Scan Asyst Fluid+ probe with a calibrated stiffness constant of k = 0.4585 N/m and a probe radius of TR = 20.3 nm was used. Local stiffness distribution maps measuring 80 × 80 μm with a resolution of 40 × 40 points were acquired. Averaging was performed over 5–6 images for each sample. The maps were analyzed with NanoScope Analysis 1.8 software.

The structure of the gel films was studied using scanning electron microscopy (Prisma E microscope, Thermo Scientific, Brno-Cernovice, Czech Republic, high vacuum mode, accelerating voltage 2–5 kV). To drain the charge when using SEM, the samples were placed on a carbon tape and a 10 nm thick gold layer was sputtered over the sample (Q150R ES, Quorum Technologies, Lewes Rd, Lewes, UK).

The presence of bound water in the structure of solid gels, which affects the rigidity of polysaccharide samples, was determined by the differential thermal analysis (DTA) of samples with a STA 449 F3 synchronous thermal analyzer from NETTZCH (GMBH, Weimar, Germany). Sample weights were 10–12 mg. The polymer destruction process was carried out in air at a gas flow rate of 20 mL/min and a linear heating rate of 10 °C/min.

Changes in the mass loss were recorded with an accuracy of 10^−3^ mg, the relative errors of temperature measurements and thermal effects were ±1.5 °C and ±3%, respectively. The destruction process was described by the dependences of the mass loss, mass loss rate, and thermal effects on the temperature. The following values were determined: the mass loss onset temperature, oxidation onset temperature, total thermal effect of the process, and maximum mass loss rate.

## 3. Results and Discussion

### 3.1. Supramolecular Structure of Alginate Matrices Obtained by Different Methods

To identify possible structural differences in alginate gels treated with calcium chloride and hydrochloric acid (that possibly affect MB immobilization and subsequent release into the external medium), atomic force and scanning electron microscopies were used. Additionally, the force volume mode of atomic force microscopy was utilized to determine the local surface stiffness (Young’s modulus).

**Figure 2:**
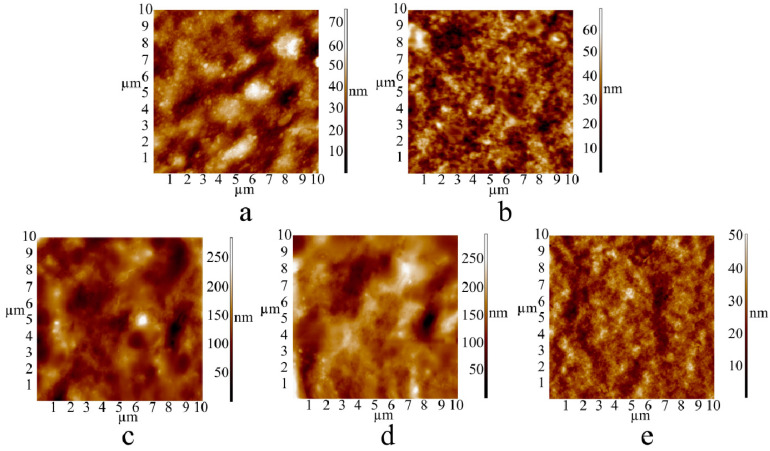
AFM images of surface areas (10 × 10 μm) of solid alginate gels treated with calcium chloride (**a**,**c**) and hydrochloric acid (**b**,**d**) in the absence (**a**,**b**) and in the presence (**c**,**d**) of PVP. SA film—(**e**).

Figure 2 shows the results of the morphology measurements of the studied surfaces of the CAG film (a), AAG film (b), CAG/PVP film (c), AAG/PVP film (d), and the SA film (e), using AFM in the topography mode.

**Figure 3:**
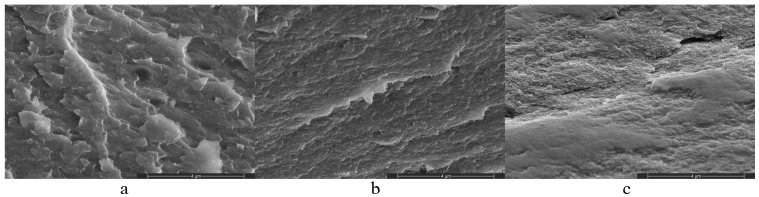
SEM images of transverse cleavages of films: (**a**) SA film, (**b**) CAG, and (**c**) CAG/PVP.

It is evident that treatment with calcium chloride (Figure 2a) leads to the development of certain structural formations on the surface areas of alginate gels, which are less pronounced in alginate matrices treated with acid (Figure 2b) [24]. It should also be noted that according to the AFM and SEM data, in compositions of alginate gels with PVP, the latter does not form a combined phase with the alginate matrix but is localized in the form of thin layers on the AAG and CAG surface and in the free volume of the polymer matrix. Since PVP is practically an amorphous polymer, its presence is recorded as a “blur” in the AFM images of the surface structure of AAG and CAG (Figure 2c,d). The AFM results are confirmed by the scanning electron microscopy data obtained from the chipped surface (Figure 3a–c), which show SEM images of the chipped surface areas of the SA film, CAG, and the CAG/PVP mixture, respectively. It is evident that treating with calcium chloride leads to the structuring of the chipped surface areas of the SA film (Figure 3a,b). As seen from Figure 3, the structure of the polymer in Figure 3a is significantly looser than that of the polymer treated with Ca^2+^ ions shown in Figure 3b. In both cases, it is evident that the polymer structure is uniform in “polymer chain packing density” throughout the entire image. The chipped surface of the SA film treated with Ca^2+^ in the presence of PVP (Figure 3c) demonstrates general “smoothness”.

**Figure 4:**
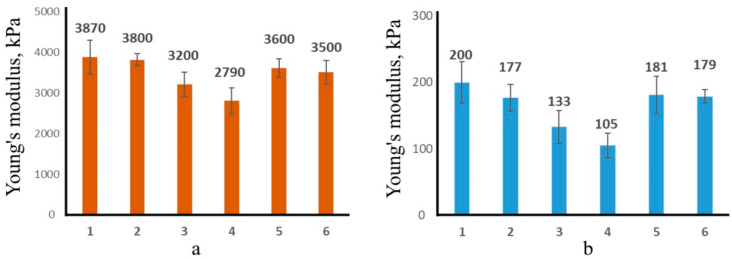
Local Young’s modulus values for Alginate Hydrogels (AHs) treated with calcium chloride (**a**) or hydrochloric acid (**b**), where: 1—AH; 2—AH/PVP; 3—AH/MB mixing; 4—AH/PVP/MB mixing; 5—AH/MB immersion; and 6—AH/PVP/MB immersion.

Thus, as follows from the AFM and SEM data, treating with calcium chloride (Figure 2a,c) leads to a more structured polymer than treating with the acid (Figure 2b,d), which should increase the local stiffness of the sample. Indeed, the measurements of the local Young’s modulus (YM) (Figure 4) showed that YM of alginate matrices treated with calcium is more than an order of magnitude higher than that of alginate matrices treated with the acid (Figure 4).

In this case, gelation in the presence of PVP led to a certain decrease in the Young’s modulus for systems treated with both calcium chloride and acid, i.e., the PVP somewhat loosens the structure of the polysaccharide. The immobilization of MB also slightly reduces the rigidity of the samples due to the fact that MB is apparently not involved in the formation of intermolecular ionic or coordination bonds in CAG.

**Figure 5:**
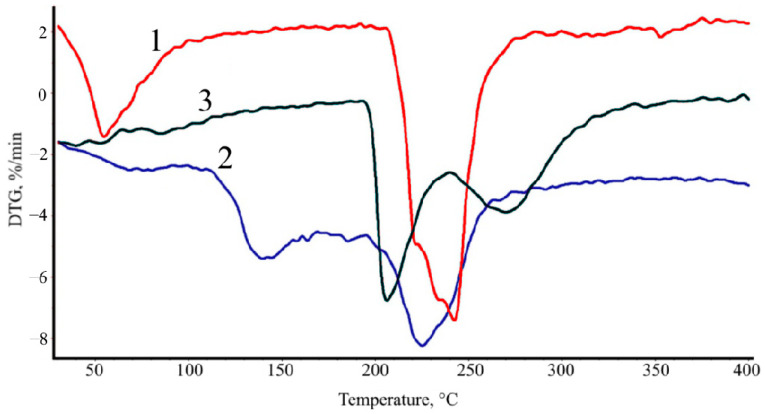
DTG curves: SA film (1), AAG (2), and CAG (3).

The formation of stronger intermolecular bonds during the formation of CAG (compared to AAG) is also confirmed by TGA. Figure 5 shows the DTG curves (dependence of the rate of mass loss on the temperature) of the SA film (1), and samples treated with calcium chloride (2) and hydrochloric acid (3).

It follows from the data presented in Figure 5 that under the conditions of thermo-oxidative destruction, water is released from the SA film at 62 °C, from acid-treated AAG at 145 °C, while from calcium chloride-treated CAG, water is released, possibly, only with the onset of the destruction of the polysaccharide macromolecules at 209 °C, as indicated by the recorded minimum of the DTG dependence. This finding indicates that in the acid-treated matrix, water molecules participate in the formation of intermolecular hydrogen bonds. In the calcium chloride-treated alginate matrix, water is probably present only in a fully “bound” state and is released during the destruction of the main chains of the polymer.

**Figure 6:**
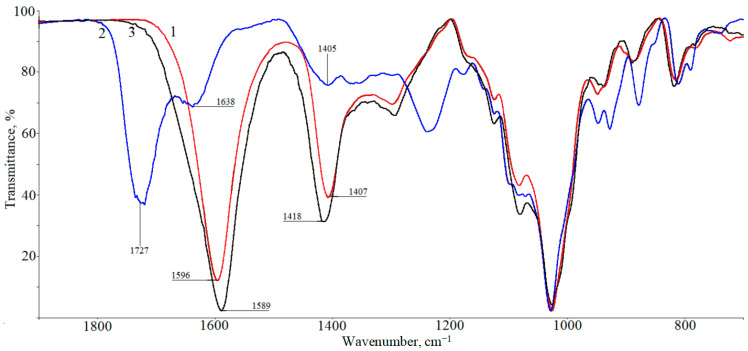
IR spectra of alginate (1), alginate treated with acid (2), alginate treated with Ca^2+^ ions (3).

Figure 6 shows the IR transmission spectra of sodium alginate (curve 1), AAG (curve 2), and CAG (curve 3). It can be seen that the spectra of the sodium alginate film (1) and SA treated with calcium ions (3) contain a band at ~1600 cm^−1^, corresponding to the vibrations of carboxylate ions (Table 1). Thus, the treatment of alginate with calcium ions has little effect on the spectral characteristics of the matrix. In addition, Figure 6 shows that in the spectrum of AAG (curve 2), a band at 1727 cm^−1^ appears, corresponding to the vibrations of undissociated carboxyl groups COOH, that indicates the formation of alginic acid. At the same time, the absorption maximum corresponding to the vibrations of carboxylate ions (1638 cm^−1^) is preserved. This finding indicates the presence of residual COO^−^ groups in the structure of AAG, and the shift in the position of the band of these groups relative to the band of carboxylate ions in the sodium alginate (1596 cm^−1^) may indicate a change in the environment of these groups (for example, the formation of hydrogen bonds with carboxyl COOH groups). Such residual carboxylate ions can obviously bind embedded methylene blue cations during MB immobilization by the immersion method, as shown below.

### 3.2. Kinetics of MB Release from Alginate Films of Different Structures into Water and Buffer Solutions

Alginic acid macromolecules, as follows from the literature, consist of residues of α-L-guluronic and β-D-mannuronic acids; moreover, in the macromolecules, as a rule, there are sections built almost exclusively from one uronic acid (G- and M-blocks) [20]. These blocks are separated by regions containing approximately equal amounts of both uronic acids, arranged in a more or less strictly alternating sequence (MG blocks) [21].

In the presence of calcium chloride, the gel is formed mainly due to an ionogenic complexation between Ca^2+^ ions and carboxylate ions. In this case, carboxylate ions, mainly of guluronic acid residues, bind to calcium ions since this is energetically more favorable in G-blocks due to the creation of spatial conditions for the strong binding of divalent metal cations.

**Figure 7:**
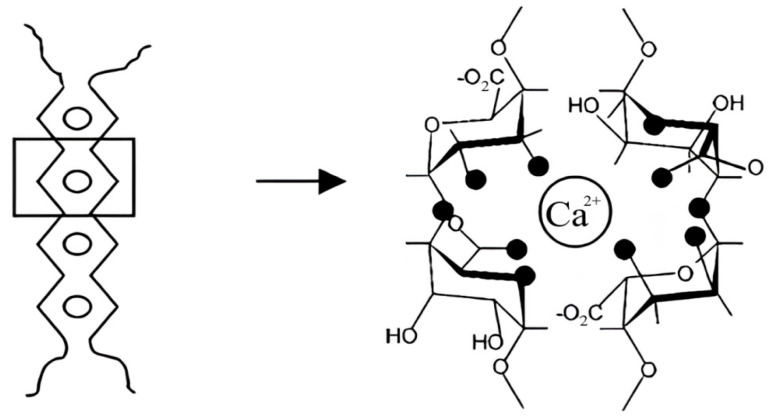
Schematic representation of calcium coordination in the eggbox model for a pair of guluronate fragments of calcium alginate macromolecules. Dark circles represent oxygen atoms involved in the coordination of the calcium ion [26,27,28].

The coordination of calcium ions by L-guluronic acid residues in the alginate molecule, illustrating the role of these ions in creating the supramolecular structure of the polymer during gelation, is shown in Figure 7 [26,27]. The mechanism of this gelation process is well known as the “egg box” model.

This model, proposed by Grant, Morris, and others in 1973 [28,29], is based on the binding of guluronate residues from two different alginate chains to the Ca^2+^ ion. These pairs, consisting of 21 helical chains, are packed with calcium ions located between them [28]. At the same time, as follows from Figure 7, some of the carboxyl and hydroxyl groups remain free. The impregnated dye can be retained in the solid gel (CAG) due to hydrogen bonds with such groups that are not involved in the binding of Ca^2+^ ions.

As already mentioned, methylene blue was introduced into the polymer matrices either at the stage of mixing, or by introducing the dye from the solution into the prepared gel. In this case, the film of the polymer was kept in a 2% aqueous solution of the dye (“immersion”). Since the activity of MB in the photogeneration of ^1^O_2_ was shown to increase in the presence of PVP [23], the samples were produced using the “mixing” and “immersion” methods, using MB in combination with PVP. Table 2, Table 3 and Table 4 show the constants *k* for the rate of MB release into water, phosphate buffer (pH 7.2), and a hydrochloric acid buffer simulating the stomach medium (pH 1.6), respectively, from solid SA gels treated with CaCl_2_ and HCl when the dye is introduced by “mixing” and “immersion”.

**Figure 8:**
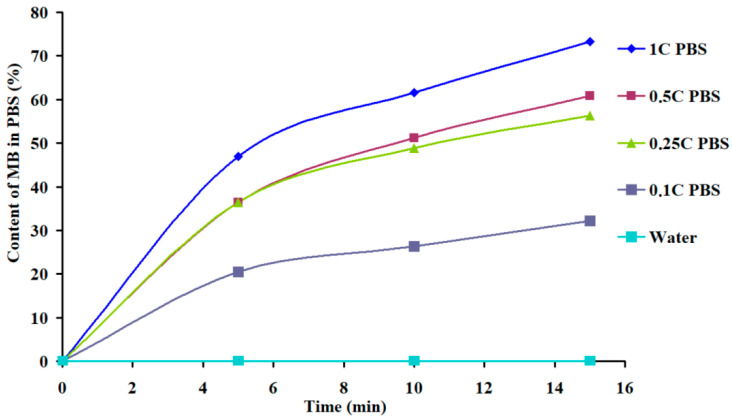
Kinetic curves of MB release from a CAG/MB sample produced by the “immerison” method into the PBS buffer with concentrations of 1C, 0.5C, 0.25C, and 0.1C, where C is 0.01 M of PBS (at pH = 7.2).

It can be seen from the data presented that MB was released into buffer solutions (at different rates) in all the cases, while into water, it is released only from systems produced by mixing, regardless of the gelation agent (Table 2, No. 5–7). From the samples produced by the “immersion” method, MB was released into the water only when introduced into a matrix treated with calcium chloride in the presence of PVP (Table 2, No. 8). It is known that the process of alginate treating with calcium chloride occurs at a high rate: the relaxation time of the slowest stage of this process is in seconds [28]. At the same time, the rate of dye interaction with the carboxyl groups of alginate should be significantly lower than the rate of a similar process of the interaction of SA with an inorganic calcium salt. In other words, it can be assumed that during dye immobilization in the process of producing the polymer system (the “mixing” stage), the connection of its molecules with the functional groups of the polysaccharide chains is due to donor–acceptor and hydrophobic interactions, as well as the forming of ionic bonds, which should ensure the maximum permissible reduction in the free energy of the system under these conditions. At the same time, while introducing cationic MB into a solid gel (i.e., the “immersion” method), when the structure of the carrier has already been formed, the MB cations should be immobilized in the form of mobile counterions in the vicinity of the carboxyl groups of SA macromolecules. As follows from the literature [27], since the interaction with the agent (in our case, with calcium chloride or hydrochloric acid) involves predominantly the carboxyl groups of the guluronic acid residues of SA macromolecules (Figure 7), some part of the carboxyl groups in the mannuronic acid blocks may remain in the original undissociated form, capable of binding methylene blue cations. The presence of dissociated and undissociated carboxyl groups in the samples treated with CaCl_2_ and HCl is indicated by the above IR spectral data (Figure 6). MB that is bound to the matrix as a counterion can leave the matrix to diffuse into the external medium (solutions with a certain ionic strength) by the ion exchange reaction with Na^+^ and K^+^ cations in the case of a phosphate buffer, and with H^+^ and K^+^ ions in the hydrochloric acid buffer medium (KCl—HCl). Indeed, the rate and amount of dye released into the solution from the matrix produced by the “immersion” method decreased with a decrease in the concentration of phosphate salts in the PBS solutions (Figure 8).

**Figure 9:**
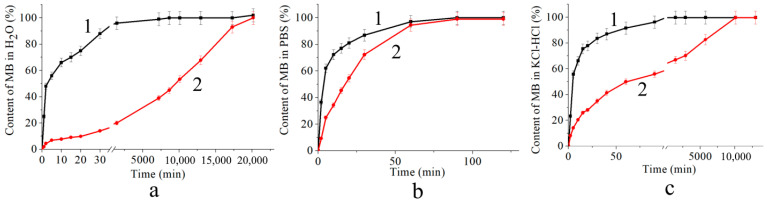
Kinetic dependences of the accumulation of MB released from the CAG (1) and AAG (2) matrices (upon introduction of the dye by the “mixing” method) into water (**a**), phosphate (pH = 7.2) buffer (**b**), and hydrochloric acid (pH = 1.6) buffer (**c**).

As can be seen from Table 2, Table 3 and Table 4 and Figure 9, the rate of MB release from the mixing-produced impregnated matrices into buffer solutions depends on the gelation agent’s nature. The constant *k* of the rate of MB release from matrices treated with calcium chloride significantly exceeds *k* of the rate of MB release from acid-treated SA matrices at the initial stage.

The same patterns are observed when MB is released from the above matrices into the acidic buffer and water. These data obviously indicate the possible partial binding of the dye with residual carboxylate ions that do not participate in the formation of the matrix structure of the alginic acid gel (AAG). Alginic acid gel is apparently formed due to the formation of intermolecular hydrogen bonds between the carboxyl (carbonyl) and hydroxyl groups of the biopolymer.

It also follows from Table 2, Table 3 and Table 4 that the rate of the release of MB introduced in the presence of PVP hardly depends on the pH of the external medium since the MB solubility in the water and buffer solutions is rather high. In this case, MB in the “external” medium is no longer in an aggregated state, as will be shown below.

### 3.3. Spectral Properties of MB in Solid Alginate Gel Matrices

**Figure 10:**
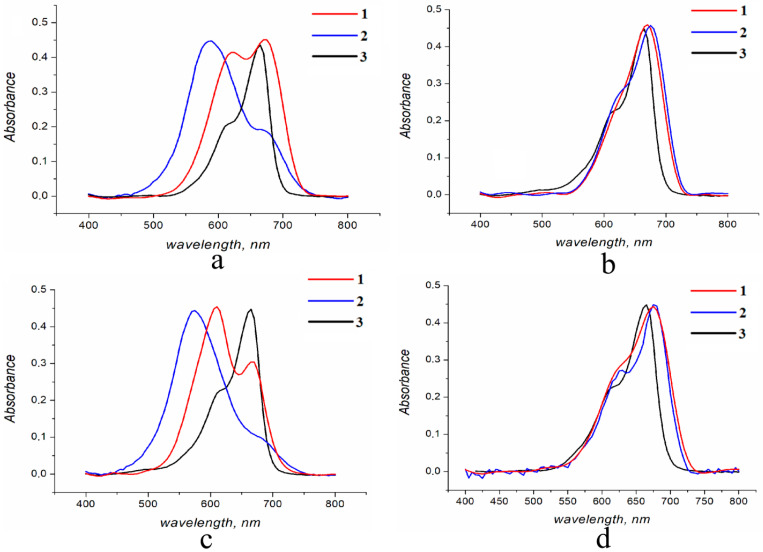
Electronic absorption spectra of MB introduced into AAG (1) and CAG (2) matrices at the stage of “mixing”, (**a**)—MB, (**b**)—MB in the presence of PVP and “immersion”, (**c**)—MB, and (**d**)—MB in the presence of PVP, as well as a solution of MB in water (3). The absorption spectra of MB in polysaccharide matrices are normalized by the optical density of the absorption band of the MB monomer in its standard aqueous solution.

The features of MB immobilization in the matrices of SA, determining the studied kinetics of MB release into aqueous media of different compositions, are clearly manifested in the study of the electronic absorption spectra of MB in such matrices. Figure 10 shows the spectra of MB introduced into SA matrices in two ways.

It is evident that when MB is introduced into the matrix at the “mixing” stage, the peak (shoulder) with λ = 615 nm in the MB spectrum grows, undergoes a hypsochromic shift, and broadens significantly. Such growth of the band corresponding to the absorption of the dimeric form of the dye (Figure 10a), the presence of which is recorded in the solution of the SA film by the corresponding shoulder [30], indicates MB aggregation. As shown earlier, amphiphilic polymers prevent the aggregation of MB molecules in aqueous solutions [23]. Apparently, similar patterns are observed for MB introduced into the SA matrix using the “mixing” method. Indeed, in Figure 10b, which shows the spectrum of the dye introduced into CAG (SA + CaCl_2_) in the presence of PVP, which prevents the ionic interaction of MB with SA, no broadening of the absorption band of the dimer and no change in the monomer–dimer ratio are observed compared to the spectrum of the original MB in water. Similar changes in the spectra of MB introduced into matrices treated with acid and calcium chloride and indicating the aggregation of MB molecules in alginate carriers are observed when introducing the dye into matrices using the “immersion” method (Figure 10c,d).

It should be noted that, after exiting the matrix into solutions with the pH range of 1.5–7.2, the ratio and position of the bands of the monomeric and dimeric forms, characteristic of the initial, non-aggregated state of the dye, are restored for MB. In addition, changing the pH of the aqueous solution in the range of 1.6–8.0 did not change the appearance of the MB electronic absorption spectrum.

Thus, the main factor affecting MB release into a medium is the method of dye immobilization. By varying the method of introducing MB, the required correlation of interactions between the MB load, the polymer gel, and external medium was achieved in order to realize the different rates of dye release into the different medium. Indeed, when the dye is introduced into the matrix at the stage of gel preparation, i.e., when treating SA with calcium chloride or hydrochloric acid, MB is released from the polysaccharide into water and into both buffers. On the other hand, MB is not released into the aqueous phase from the samples obtained by introducing MB into fully formed gels, where the film is kept in the dye solution but is released in an acidic or alkaline buffer. In other words, this means that the dye is fixed in the matrix in different charged states. Apparently, when introduced into the matrix at the “mixing” stage, MB is immobilized in its molecular form and binds to the matrix due to weak donor–acceptor interactions. This is indicated by a significantly higher (by an order of magnitude) value of the rate constant of MB release (into all media from alginate matrices treated with calcium chloride when introducing the dye at the “mixing” stage) compared to the rate constant of MB release from the same matrices prepared by the “immersion” technique. Therefore, it can be assumed that at the “mixing” stage, MB is fixed in the matrix as a molecule, and during “immersion” it is fixed as an ion, since in this case, MB is released only into a salt solution (acidic or slightly alkaline).

## 4. Conclusions

Solid gels based on calcium alginate (CAG) and alginic acid (AAG) have been developed, containing an efficient photosensitizer of singlet oxygen generation, methylene blue (MB). The kinetics of its release from the gels has been studied. The solid gels were prepared by the interaction of calcium chloride and hydrochloric acid with sodium alginate. The dye was introduced into the matrices either at the stage of gelation (mixing), or by the immersion of the hydrogel into an aqueous solution of the dye (immersion). It has been shown that the strength of the dye’s attachment to the AAG matrix is higher than that of the CAG, which leads to a higher rate of MB release from CAG into aqueous media. It has been shown that when introduced by the immersion method, the dye may be localized in the matrix as a fixed counterion of the carboxyl groups in AAG and CAG. When introduced by the mixing method, MB is retained in AAG or CAG as a mobile counterion. In the latter case, its release into the external medium is facilitated. In the case of the dye’s localization in the matrix in the form of a fixed counterion, MB may be released only into salt solutions. Such a system—MB immobilized on an alginate gel—may be used as a solid photosensitizing system with the controlled release of the photoactive agent into the wound cavity for the photodynamic treatment of purulent wounds, bedsores, and trophic ulcers.

## Figures and Tables

**Table 1:** Assignment of the main bands in the IR spectra of alginate-based systems [25].

Bands	Assignment
1727	COOH
1638; 1589−1596; 1405−1418	COO^−^
1200−1420	CO, OH, CH

**Table 2:** Kinetic parameters of the diffusion process of MB from the formed matrices into water.

Sample	Sample Number	50% Release	k × 10^3^, s^−1^
Mixing			
AAG/MB	1	7 days	0.8
CAG/MB	2	2 min	10.0
AAG/PVP/MB	3	180 min	1.3
CAG/PVP/MB	4	2 min	7.7
Immersion			
AAG/MB	5	No release	
CAG/MB	6	No release	
AAG/PVP/MB	7	No release	
CAG/PVP/MB	8	30 min	1.2

**Table 3:** Kinetic parameters of the diffusion process of MB from the formed matrices into PBS, pH = 7.2.

Sample	Sample Number	50% Release	k × 10^3^, s^−1^
Mixing			
AAG/MB	9	20 min	0.7
CAG/MB	10	3 min	8.2
AAG/PVP/MB	11	40 min	0.8
CAG/PVP/MB	12	3 min	6.9
Immersion			
AAG/MB	13	5 min	1.2
CAG/MB	14	5 min	7.9
AAG/PVP/MB	15	25 min	0.6
CAG/PVP/MB	16	30 min	0.5

**Table 4:** Kinetic parameters of the diffusion process of MB from the formed matrices into the acidic medium KCl-HCl, pH = 1.6.

Sample	Sample Number	50% Release	k × 10^3^, s^−1^
Mixing			
AAG/MB	17	180 min	0.8
CAG/MB	18	5 min	10.0
AAG/PVP/MB	19	3 min	9.0
CAG/PVP/MB	20	15 min	10.0
Immersion			
AAG/MB	21	5 min	4.9
CAG/MB	22	5 min	5.9
AAG/PVP/MB	23	5 min	7.0
CAG/PVP/MB	24	12 min	6.0

## Data Availability

The data presented in this study are available on request from the corresponding author.
